# Diagnosis and management of arrhythmias in dogs: A cross‐sectional online survey among Flemish veterinary practitioners

**DOI:** 10.1002/vro2.35

**Published:** 2022-04-18

**Authors:** Arnaut Hellemans, Mark Schittekatte, Marc Covents, Pascale Smets

**Affiliations:** ^1^ Small Animal Department Faculty of Veterinary Medicine Ghent University, Salisburylaan 133 Merelbeke Belgium; ^2^ Research Support Office Faculty of Psychology and Educational Sciences Ghent University, Henri Dunantlaan 2 Ghent Belgium

## Abstract

**Background:**

Diagnosis as well as management of arrhythmias in dogs can be challenging for veterinary practitioners. The aim was to describe ECG availability and use, as well as the diagnostic and therapeutic experiences and preferences of Flemish veterinarians regarding cardiac arrhythmias in dogs.

**Methods:**

Cross‐sectional online survey among veterinarians in Flanders (Belgium).

**Results:**

An ECG device was available for 55 out of 102 respondents (54%) and 41 (43%) claimed to use it in case of arrhythmia suspicion. Insufficient knowledge about ECG interpretation and immediate patient referral upon detection of an abnormal heart rhythm were the most important reasons for not having, or not using, an ECG. About half of the respondents (56%) had never used anti‐arrhythmic drugs in dogs, although only a few reported having had a negative experience. Frequently provided reasons for not using anti‐arrhythmic drugs included insufficient knowledge and a low number of dogs with arrhythmias.

**Conclusion:**

Most veterinarians reported having little or no expertise with arrhythmias in dogs. Electrocardiogram availability and use among respondents was moderate and too often restricted by insufficient ECG interpretation skills. Continued efforts are needed to increase the confidence and knowledge of veterinarians about arrhythmias in dogs.

## INTRODUCTION

Cardiac arrhythmias have been reported in 3.2% of dogs in a general referral population and up to 39.5% in dogs referred for cardiac and ECG evaluation.^1^, ^2^ However, the true prevalence of cardiac arrhythmias in the general canine population remains unknown and is likely underestimated. In both dogs and humans, arrhythmic heart disease has been shown to often remain clinically silent for years, until sudden death or heart failure may occur.^3^, ^4^, ^5^ Because of the paroxysmal nature of many arrhythmias, obtaining an ECG recording during the event is crucial. The role of prolonged ECG monitoring and additional ECG screening by the general practitioner has been of increasing interest in human medicine.^6^ Likewise, general veterinary practitioners could play a role in early detection of cardiac arrhythmias in dogs prior to referral. This emphasises the importance of theoretical knowledge and awareness about cardiac arrhythmias in dogs among veterinarians and highlights the need for the presence of basic diagnostic equipment such as an ECG recorder.

The primary aim of this exploratory study was to describe the attitude of veterinarians towards arrhythmias in dogs. We sought to estimate the availability, use and frequency of use (per month) of ECG recorders. Furthermore, we aimed to investigate diagnostic preferences in case of arrhythmia suspicion in a dog. In addition, we sought to evaluate the use of and experiences with anti‐arrhythmic drugs among respondents. The secondary aim was to identify factors restricting ECG use and availability, as well as anti‐arrhythmic drug use in dogs.

## MATERIAL AND METHODS

### Respondents

A cross‐sectional online survey study was conducted in Flanders and Brussels‐Capital region (Belgium). The target group included all veterinarians working part time or full time as veterinary practitioner, including mixed practice for small and large animals and university. Respondents with a degree in veterinary medicine who were not working as veterinary practitioner and veterinarians working outside the target region, were excluded. There were no minimum requirements regarding cardiology workload. Given the variety of additional courses and training among the respondents, we did not distinguish between different levels of specialisation. Any members of the European Board of Veterinary Specialisation (EBVS), specifically the European College of Veterinary Internal Medicine (subspecialty cardiology), were excluded because their level of expertise would not reflect the general veterinary population.

### Data collection

Responses were collected over a fixed 6‐week period, between January and February 2021. The survey was actively distributed and advertised via veterinary professional associations (SAVAB Flanders and VeDa), the ‘Flemish Veterinary Journal’, the veterinary magazine ‘Dierenartsenwereld’, social media and LinkedIn. Both online and printed advertisements were used to maximise the response rate. In addition, individual e‐mail invitations were sent to members of the previously mentioned veterinary associations via a monthly newsletter with a reminder 1 week before the end of the survey. Participation was voluntary and data were gathered based on individual informed consent, compliant with the EU General Data Protection Regulation and data management requirements of Ghent University. Veterinarians that wished to participate were invited to visit the survey website providing all necessary information regarding participant rights, future data use and survey goal. By clicking the ‘participate’ button, respondents agreed to the terms of use and were taken directly to the online questionnaire created by LimeSurvey (LimeSurvey GmbH).

### Survey

The survey consisted of 49 close‐end questions in Dutch. Depending on the type of question, two or more answers could be selected from a dropdown menu. The questionnaire was further divided in four main sections. Section 1 asked for age category, gender, diploma, time since graduation, current employment as veterinary practitioner, region of employment and type of veterinary practice. For privacy reasons, most personal data were requested as categories or intervals instead of exact numerical data. Section 2 inquired about expertise in cardiology and cardiac arrhythmias. Respondents were asked if they had taken one or more courses in cardiology or if they had obtained additional certificates or degrees in cardiology. This section also included two subjective self‐assessment scores concerning expertise in general cardiology and cardiac arrhythmias in dogs using a five‐point Likert‐scale (no expertise = 1, very good expertise = 5).

Section 3 contained questions regarding the presence of diagnostic equipment (ECG and 24‐hour Holter ECG), the clinician's attitude towards different diagnostic modalities and the use and estimated frequency of use of this diagnostic equipment in dogs. Frequencies were requested as rate‐based estimation (frequency in a typical month).^7^ Respondents who did not have or did not use an ECG or 24‐hour Holter recorder were asked about the main underlying reason. Respondents were also asked to select all arrhythmias that they had diagnosed in the past in their practice. The fourth section concerned the clinician's use of and experience with anti‐arrhythmic drugs. A list of drugs with anti‐arrhythmic properties was presented and participants were asked to select up to three drugs that they had used in their practice. Veterinarians who did not use anti‐arrhythmic drugs were asked for the main underlying reason. Finally, we asked respondents how they rated treatment effect in dogs and whether they encountered a situation where they had to stop treatment early.

### Data analysis

The complete dataset was extracted as a Microsoft Excel file from LimeSurvey and entered in SPSS 27 statistics (IBM) for descriptive analysis. A Wilcoxon signed rank test was used to compare expertise in general cardiology with expertise in cardiac arrhythmias in dogs. Frequency distributions and percentages were calculated and tabulated for the general veterinary population and the subpopulations of veterinarians working primarily in private practice or working at a university. We reasoned this could enhance future comparison against similar surveys in other countries or regions, where the respondent population may be different from ours. It was, however, neither our aim nor feasible (given the small number of people from university) to investigate or speculate on differences between the two groups.

## RESULTS

A total of 150 responses were received between January and February 2021. Given that only one response was obtained from the Brussels‐Capital Region, only responses from Flanders were considered. Sixteen responses were removed because of incomplete answers sets. Thirty‐one responses were removed because inclusion criteria were not met (n = 4, no veterinary diploma; n = 6, not practising veterinary medicine; n = 21, working outside of Flanders or Brussels‐Capital Region). A total of 102 valid responses remained. Due to the open sampling method no response rate could be calculated.

### Demographics

Table 1 shows the self‐reported characteristics of the 102 Flemish respondents and is further divided into veterinarians working primarily in private practice or in a university setting. Overall, more female (86/102) than male (16/102) respondents were present in our study sample. The majority of respondents obtained their diplomas less than 20 years ago. Most respondents (82/102) were employed in companion animal group practices. All five Flemish provinces were represented in the survey.

**TABLE 1:** Self‐reported demographics of the 102 respondents who completed the online survey

			Subpopulation	
	% (all practitioners)	*n* (all practitioners)	*n* (private practice)	*n* (university)
Sex				
Female	84.3	86	69	17
Male	15.7	16	14	2
Time since graduation				
<10 years	61.8	63	51	12
11–20 years	27.5	28	22	6
21–30 years	7.8	8	7	1
31–40 years	2.9	3	3	0
Subspecialisation (Master degree)				
Companion animals	80.4	82	64	18
Ruminants	5.9	6	6	0
Horses	10.8	11	11	0
Research	1.0	1	0	1
General diploma	2.0	2	2	0
Type of veterinary practice				
Single person practice	19.6	20	20	0
Group practice (two or more)	56.9	58	58	0
University	18.6	19	0	19
Mixed	3.9	4	4	0
Other	1.0	1	1	0
Region of employment				
Province of Antwerp	24.5	25	24	1
Province of Limburg	12.7	13	13	0
Province of East Flanders	35.3	36	18	18
Province of Flemish Brabant	12.7	13	13	0
Province of West Flanders	14.7	15	15	0

### Expertise

In the first part of the questionnaire, respondents were objectively and subjectively assessed for their expertise in cardiology and arrhythmias in dogs. Table 2 displays a summary of the results. Most (86/102) had followed one or more cardiology courses in the past 5 years. A small number (5/102) had obtained an additional degree or certificate in cardiology. Regarding cardiology in dogs, the majority rated their expertise as lower than or equal to moderate. Expertise in arrhythmias in dogs was rated significantly lower than expertise in cardiology (Wilcoxon signed rank test, *p* < 0.001), with approximately two‐thirds rating their expertise as little or none.

**TABLE 2:** Self‐rated expertise in cardiology and cardiac arrhythmias in dogs among 102 respondents

			Subpopulation	
	% (all practitioners)	*n* (all practitioners)	*n* (private practice)	*n* (university)
Followed one or more cardiology courses (<5 years)				
Yes	66.7	68	60	8
No	33.3	34	23	11
Obtained additional cardiology certificate				
Yes	4.9	5	4	1
No	95.1	97	79	18
Expertise in cardiology in dogs				
None	3.0	3	3	0
Little	33.3	34	27	7
Moderate	40.6	41	35	6
Good	19.8	20	14	6
Very good	3.0	3	3	0
Skipped question		1		
Expertise in cardiac arrhythmias in dogs				
None	12.7	13	13	0
Little	52.0	53	44	9
Moderate	28.4	29	21	8
Good	6.9	7	5	2
Very good	0.0	0	0	0

### Diagnosis

Numerical data on the availability and use of diagnostic ECG equipment, diagnostic preferences and self‐reported arrhythmia observations in dogs are displayed in Table 3. Concerning the presence of diagnostic equipment, 55 out of 102 respondents had access to an ECG device. When present, 37 out of 55 respondents used it (see Table 3 for usage frequencies). A 24‐hour Holter device was accessible by 21 out of 102 respondents and 5/21 used this device. The most common reason for not having an ECG device was little or no expertise in ECG interpretation (24/47). This was followed by respondents that were employees unable to decide over the purchase of an ECG device (13/47), respondents with a low number of dogs with arrhythmias (5/47), respondents that were put off by the purchase price (2/47) and respondents that had a defective device (1/47). The most frequent reasons for not having a 24‐hour Holter ECG included little or no expertise in Holter ECG interpretation (31/81) and case referral (21/81). Respondents that did not to use an ECG device even though it was available, reported their main reasoning as patient referral (11/17). Other reasons included little or no expertise in ECG interpretation (5/17) and a low number of dogs with arrhythmias (1/17).

**TABLE 3:** Availability, use and frequency of use of diagnostic ECG equipment, diagnostic preferences and self‐reported arrhythmia observations in dogs

			Subpopulation	
	% (all practitioners)	*n* (all practitioners)	*n* (private practice)	*n* (university)
Is an ECG recorder or Holter available on site?				
ECG present	53.9	55	39	16
Holter device present	20.6	21	7	14
Do you use an ECG recorder in dogs yourself?				
Yes	67.3	37	26	11
No	32.7	18	13	5
Skipped question		47		
Do you use a 24‐hour Holter recorder in dogs yourself?				
Yes	23.8	5	3	2
No	76.2	16	4	12
Skipped question	81			
How frequently do you use this ECG device in dogs in a typical month? (excluding anaesthetic monitoring)				
<1x/month	40.5	15	13	2
1–2x/month	24.3	9	6	3
3–4x/month	18.9	7	5	2
>4x/month	16.2	6	2	4
Skipped question		7		
What is the main reason you do not possess an ECG?				
Purchase price is too high	4.3	2	2	0
No or too few dogs with arrhythmias	10.6	5	5	0
Little or no expertise in ECG interpretation	51.1	24	23	1
Device is defective	2.1	1	1	0
I do not decide this myself	27.7	13	12	1
None of the above	4.3	2	1	1
Skipped question		55		
What is the main reason you do not use an ECG in dogs?				
No or too few dogs with arrhythmias	5.9	1	1	0
Little or no expertise in ECG interpretation	29.4	5	5	0
Patient referral	64.7	11	6	5
Skipped question		85		
Which diagnostic steps do you take if a cardiac arrhythmia is suspected in a dog based on auscultation?				
Electrocardiography	43.2	41	27	14
Echocardiography	20.0	19	16	3
Blood examination	20.0	19	14	5
Chest radiographs	29.5	28	24	4
Repeat auscultation (at later timepoint)	30.5	29	24	5
Patient referral	74.7	71	57	14
Skipped question		7		
Have you suspected an arrhythmia in a dog based on auscultation?				
Yes	93.1	95	76	19
No	6.9	7	7	0
Have you diagnosed an arrhythmia in a dog based on ECG?				
Yes	42.2	43	28	15
No	57.7	59	55	4
Which of the following arrhythmias have you diagnosed in the past in a dog?				
Atrial fibrillation	71.4	25	17	8
Supraventricular premature complexes	40.0	14	9	5
Supraventricular tachycardia	28.6	10	5	5
Ventricular premature complexes	71.4	25	15	10
Ventricular tachycardia	51.4	18	13	5
Atrioventricular block grade II	60.0	21	15	6
Atrioventricular block grade III	28.6	10	6	4
Sinus bradycardia	34.4	12	5	3
Sick sinus syndrome	14.3	5	2	3
Skipped question		67		

Most frequent choices for the diagnostic investigation of dogs with suspected arrhythmias included referral (71/95), ECG (41/95), thoracic radiographs (28/95), echocardiography (19/95) and blood examination (19/95).

Nearly all respondents (95/102) had suspected a cardiac arrhythmia in a dog in the past based on heart auscultation. Most commonly diagnosed arrhythmias included atrial fibrillation (25/35), ventricular premature complexes (25/35), atrioventricular block grade II (21/35), ventricular tachycardia (18/35) and supraventricular premature complexes (14/35).

### Treatment

Table 4 displays numerical data on the use of anti‐arrhythmic drugs and therapeutic preferences in dogs among 102 respondents. About half of the respondents (57/102) answered they had never administered, prescribed or provided anti‐arrhythmic drugs to initiate or continue a treatment in dogs. Frequently provided underlying reasons included insufficient knowledge about these drugs (37/57) and a low number of dogs with arrhythmias (11/57) in their practice.

**TABLE 4:** Use of anti‐arrhythmic drugs in dogs and therapeutic preferences among 102 respondents

			Subpopulation	
	% (all practitioners)	*n* (all practitioners)	*n* (private practice)	*n* (university)
Have you ever administered, prescribed or provided anti‐arrhythmic drugs in the past to initiate or continue treatment in dogs with cardiac arrhythmias?				
Yes	44.1	45	35	10
No	55.9	57	48	9
What is the main reason for not prescribing or administering anti‐arrhythmic drugs to dogs?				
No or too few dogs with arrhythmias	19.3	11	11	0
Dog is showing little or no signs	1.8	1	1	0
Insufficient knowledge about these drugs	64.9	37	31	6
None of the above	14.0	8	5	3
Skipped question		45		
Which of the following anti‐arrhythmic drugs have you used in the past? You may indicate up to three drugs?				
Amiodarone	2.2	1	1	0
Atenolol	51.1	23	20	3
Digoxin	46.7	21	20	1
Diltiazem	46.7	21	15	6
Esmolol	4.4	2	0	2
Lidocaine	31.1	14	5	9
Magnesium	4.4	2	2	0
Mexiletine	2.2	1	0	1
Propranolol	4.4	2	2	0
Sotalol	31.1	14	11	3
None of the above	4.4	2	2	0
Skipped question		57		
What do you rely on most to evaluate the therapeutic effect of anti‐arrhythmic drugs in dogs?				
Clinical examination	20.0	9	9	0
Control ECG	37.8	17	12	5
Control Holter ECG	17.8	8	6	2
I do not evaluate the effect	20.0	9	6	3
None of the above	4.4	2	2	0
Skipped question		57		
What is the main reason if you have to stop anti‐arrhythmic drug treatment in dogs?				
Adverse effects	55.6	5	3	2
Insufficient clinical improvement	33.3	3	1	2
Owner unwilling or unable to administer medication	11.1	1	1	0
Skipped question		93		

When asked to select up to three anti‐arrhythmic drugs which they had used in the past, most frequent responses included atenolol (23/45), digoxin (21/45), diltiazem (21/45) and lidocaine (14/45). Some respondents answered esmolol (2/45), magnesium (2/45), propranolol (2/45), amiodarone (1/45) or mexiletine (1/45).

Some respondents (9/45) that had used these drugs in their practice declared that they had encountered a situation where they had to stop anti‐arrhythmic treatment. Reasons included suspected adverse reactions (5/9), insufficient clinical improvement (3/9) and unwillingness or inability of the owner to give oral medications (1/9).

Respondents were also asked how they evaluated treatment success with anti‐arrhythmic drugs in dogs, by choosing the most important parameter from a list. The most frequent answers included an ECG (17/45), followed by a physical examination (9/45) and a Holter examination (8/45).

## DISCUSSION

This study, to the best of the authors’ knowledge, is the first report on the availability and use of ECGs and attitude of veterinarians towards the diagnosis and treatment of cardiac arrhythmias in dogs. An important finding was the moderate use and availability of ECGs among respondents, which was often related to insufficient ECG interpreting skills. About half of the respondents had no experience of treating dogs with arrhythmias using anti‐arrhythmic drugs. Respondents attributed this to a lack of knowledge about these drugs and the low number of dogs with arrhythmias in their practice.

It is of concern that 66 of the 102 veterinarians judged their expertise in arrhythmias in dogs as little to none. Insufficient ECG interpreting skills was a common answer to several questions in this survey. Previous studies in human medicine have revealed moderate ECG interpreting competences among medical personal.^8^, ^9^, ^10^ In one study, about one‐third of general practitioners and nurses felt very to fairly uncomfortable interpreting a routine ECG.^9^ Despite the fact that our study was not aimed at scoring ECG competences across veterinarians, our findings suggest there should be further investigation. Basic ECG interpretation competency is part of the veterinary master programme in Belgium. Additionally, almost all participants had followed some cardiology‐related continuing education in the past 5 years. However, it is well known that correct ECG interpretation requires a substantial amount of repeated practical training. A study in human medicine among more than 300 first‐year medical students showed that a diagnostic accuracy of 85% could only be achieved after 73 cases and >200 minutes training.^11^ Thus, the question arises as to whether it was realistic to expect our respondents to feel comfortable in ECG interpretation with less than the above‐mentioned training.

Availability of ECG devices among respondents was moderate. An important reason for not having or using an ECG was a lack of ECG interpretation skills. In those cases where an ECG device was available, only a minority used it more than twice per month. It would have been interesting to know if these respondents with more frequent ECG use just had a higher cardiology workload or were motivated to do so because of other reasons. Unfortunately, no question regarding cardiology workload was included.

When asking respondents for their diagnostic approach in case of arrhythmia suspicion in a dog, the answers in order of decreasing frequency were: patient referral, an ECG recording, repeated cardiac auscultation at a later time point, thoracic radiographs, echocardiography and blood examination. Patient referral is an appropriate response, especially in case one does not have an ECG or does not feel comfortable interpreting it. However, the fact that an ECG was only considered by 43% of respondents in case of arrhythmia suspicion could have clinical consequences. For example, normal auscultation findings may be present at the time of referral due to its intermittent nature, potentially leading to higher costs for more extensive examinations such as a 24‐hour Holter ECG and echocardiography. Furthermore, not all owners will accept the offer of referral based on an irregular auscultation. Documenting the arrhythmia with an ECG recording, even if one is not able to interpret it immediately oneself, is therefore useful according to the authors. In this regard, tele‐ECG interpretation services could theoretically offer substantial benefits for both veterinarian, dog and owner.

This study also provided a list of frequently encountered arrhythmias by veterinary practitioners in Flanders. The question was not limited to primary arrhythmias and could include secondary arrhythmias observed during anaesthesia or during a post‐operative hospitalisation period. These data should not be confused with the prevalence of arrhythmias in dogs in Flanders, which is unknown. The two most observed arrhythmias were atrial fibrillation and ventricular premature complexes. This makes sense as both have been reported to have the highest prevalence of all pathological arrhythmias in dogs.^2^, ^12^ Other frequently encountered arrhythmias included atrioventricular block grade II, ventricular tachycardia and supraventricular premature complexes.

As for anti‐arrhythmia therapy, more than half of the respondents had never used anti‐arrhythmic drugs in dogs with arrhythmias. Insufficient knowledge about these drugs was the most common reason, provided by two‐third, followed by respondents who had too few dogs with arrhythmias in their practices. However, it is important to bear in mind that 59 out of 102 never diagnosed an arrhythmia. In addition, not all arrhythmias require anti‐arrhythmic treatment and correct identification and risk stratification should always take place. Because we have no information on the cardiology workload of our respondents and do not know if they ever diagnosed an arrhythmia in a dog that also required treatment, no firm conclusion can be drawn here.

Respondents who answered yes to the previous question ‘Have you ever administered, prescribed or provided anti‐arrhythmic drugs in the past to initiate or continue treatment in dogs with cardiac arrhythmias?’ were asked to select up to three drugs with anti‐arrhythmic properties that they had used in the past. We acknowledge that this follow‐up question could have confused some respondents, as we did not specifically ask if in those instances they were used in dogs and for the indication of an arrhythmia. This could partly help to explain why the most commonly reported drug was the beta‐blocker atenolol, which in addition to being an anti‐arrhythmic is often used for other indications (e.g. in stenotic heart disease and obstructive hypertrophic cardiomyopathy).^13^, ^14^, ^15^ The frequent use of digoxin and diltiazem could be due to their use as first‐line treatment for atrial fibrillation, which was the most commonly diagnosed arrhythmia among the respondents.^16^ Similarly, the relatively large number of respondents who used lidocaine and sotalol could be explained by the fact that ventricular tachycardia was the fourth most diagnosed arrhythmia in this study. Both sotalol and lidocaine are described a first‐line treatment for ventricular tachycardia.^17^ We also tried to speculate a few potential reasons why amiodarone, mexiletine, magnesium and esmolol were used so infrequently. For example, amiodarone is considered a second‐line drug for atrial fibrillation^16^ and ventricular arrhythmias.^17^ Mexiletine has availability issues in Europe and is expensive for long‐term treatment. For other drugs such as magnesium and esmolol, there is still uncertainty about their efficacy and their specific indications.^18^, ^19^

Respondents that used anti‐arrhythmic drugs and evaluated the treatment effect relied mostly on ECG, physical examination or Holter ECG. The popularity of a standard ECG is likely due to practical reasons such as availability. Nonetheless, the diagnostic superiority of a 24‐hour Holter ECG recording over a short ECG has been proven for atrial fibrillation.^20^ Lastly, a significant portion did not evaluate the therapeutic effect which is rarely advisable given the potential pro‐arrhythmic effect of many of these drugs.

The first limitation of our study was the absence of a response rate. The National Veterinary Association or ‘Nederlandstalige Gewestelijke Raad van de Orde der Dierenartsen' could not provide numbers on active veterinary practitioners in Flanders. A request to e‐mail the questionnaire to a randomised sample of all active veterinary members was not accepted. In order to maximise the study sample, our study used the website, mailing list and magazine of several veterinary professional organisations. We can assume we did not reach all veterinarians working in Flanders, as not all are member of a veterinary association or accessed our social media or LinkedIn during the duration of survey. Therefore, we cannot exclude that membership to one of the previously mentioned organisations, magazines and journal influenced our results. Given that participation was voluntary, we can also not exclude the presence of a self‐selection bias, meaning we attracted more veterinarians with an interest in this topic. Unfortunately, no question related to cardiology workload was included in the survey. Considering our small non‐randomised sample size, a certain degree of caution must be applied, as the findings might not be representative for the Flemish veterinary population. Most veterinarians in our study sample were female, specialised in companion animals and employed in a group practice. These findings were in line with an earlier survey among Flemish veterinarians.^21^ Nineteen out of 102 respondents worked as a veterinarian in a university setting and this will have influenced their region of employment. Nevertheless, all five provinces were represented in the survey sample. The authors chose to retain these veterinarians working in a university setting as part of the general veterinary population and include them in the main analysis. The rationale behind this was that they were not members of the EBVS and it is common in Belgium that they work part time in private practice. Lastly, the results of this study may not reflect the situation in other countries, which would require a larger multinational randomised cross‐sectional survey.

In conclusion, our findings highlight that a significant proportion of veterinarians in Flanders did not have an ECG recorder in their practice or did not use it in dogs. All too often, availability and use were limited by insufficient ECG interpretation skills of the respondent. Instead, patient referral was the most frequent choice upon detection of an abnormal heart rhythm in a dog. The use of anti‐arrhythmic drugs in dogs appeared to be infrequent. Insufficient knowledge about these drugs was an important factor preventing a more widespread use. Taken together, more efforts should be made to increase the confidence of veterinarians regarding the diagnosis and management of cardiac arrhythmias in dogs, in order to improve early detection and management. Additionally, promotion of ECG interpretation by tele‐ECG services may be an incentive for more veterinarians to record ECGs themselves.

## FUNDING INFORMATION

Flemish Research Foundation, grant number 1S78821N.

## CONFLICTS OF INTEREST

The authors declare they have no conflicts of interest.

## ETHICS STATEMENT

Ethical approval was not required under the Belgian law of 7 May 2004 concerning experiments on human subjects due to anonymous data collection and processing and informed consent.

## Data Availability

The data that support the findings of this study are available on request from the corresponding author. The data are not publicly available due to privacy or ethical restrictions.
